# Enhancement of protective vaccine-induced antibody titer to swine diseases and growth performance by Amino-Zn, yucca extract, and β-mannanase feed additive in wean-finishing pigs

**DOI:** 10.3389/fvets.2023.1095877

**Published:** 2023-08-15

**Authors:** Vetriselvi Sampath, Sungbo Cho, Byung Ryol Lee, Nam-Hun Kim, In Ho Kim

**Affiliations:** ^1^Department of Animal Resources, Dankook University, Cheonan, Republic of Korea; ^2^ZinexBio Corporation, Asan, Republic of Korea

**Keywords:** Amino-Zn, *Yucca schidigera* extract, β-mannanase, antibody, vaccination, pigs

## Abstract

The primary purpose of this research is to determine the effect of Amino-Zn (AZn), *Yucca schidigera* extract (YE), and β-mannanase enzyme supplementation on growth performance, nutrient digestibility, fecal gas emission, and immune response in pigs. A total of 180 crossbred pigs (6.57 ± 1 kg) were randomly assigned to one of three dietary treatments: CON-corn soybean meal (basal diet); TRT1-CON +1,000 ppm AZn + 0.07% yucca extract (YE) + 0.05% β-mannanase; and TRT2-CON +2,000 ppm AZn + 0.07% YE+ 0.05% β-mannanase for 22 weeks. Each treatment had 12 replicates with 5 pigs per pen. Pigs fed a diet supplemented with AZn, YE, and β-mannanase linearly increased (*p* < 0.05) BW and average daily gain at weeks 6, 12, 17, and 18. In contrast, the gain-to-feed ratio showed a linear increase (*p* < 0.05) from weeks 6 to 17 and the overall trial period. Moreover, the inclusion of experimental diets linearly decreased (*p* > 0.05) noxious gas emissions such as ammonia, hydrogen sulfide, acetic acid, carbon dioxide, and methyl mercaptans. The dietary inclusion of AZn, YE, and β-mannanase significantly increased the serological immune responses to *Mycoplasma hyopneumoniae* (MH) and foot-and-mouth disease virus (FMDV-O type) at the end of week 6 and porcine circovirus-2 (PCV-2) at week 19. Based on this result, we infer that the combination of AZn, YE, and β-mannanase supplement would serve as a novel in-feed additive to enhance growth performance and act as a boosting agent and immune stimulatory to increase the efficacy of swine vaccinations.

## 1. Introduction

It has been well documented that weaning is the most complicated part of pig production. During weaning, piglets may be exposed to various stressors, such as introducing new litter mates and a change of diet from liquid to solid, which leads to disruption of intestinal flora disorder, diarrhea, declined growth performance, preweaning diarrhea, and mortality (1). These problems represent a substantial economic loss to the livestock industry. In this regard, plants and their extracts play a vital role in promoting animal growth, enhancing immunity, and maintaining gut health (2, 3). Notably, *Yucca schidigera* extract (YE), a natural plant product derived from the *Yucca schidigera* plant, native to Southern California and Mexico, contains insoluble fibers, and high cellulose helps to improve feed efficiency and increases poultry production (4). Previously, Sahoo et al. (5) reported that YE additives significantly improved the growth performance of broilers. For more than 30 years, YE has been used to reduce ammonia and improve the biodegradation of animal waste (6). Moreover, β-Mannan, an anti-nutritional factor found in soybean meal (SBM), has also received much attention in swine nutrition (7). For instance, Kim et al. (8) reported that the graded level (400–1,600 U/kg) of β-mannanase supplementation improved the performance of growing pigs. Similarly, a study by Pettey et al. (9) demonstrated that pigs fed a corn–SBM diet supplemented with 0.05% β-mannanase improved the growth performance of wean-finishing pigs with minimal effects on nutrient digestibility. In addition, Jo et al. (10) concluded that 0.05% α-amylase + β-mannanase + protease blends improved the growth performance of growing pigs. On the other hand, Upadhaya et al. (11) reported that a corn–SBM diet supplemented with β-mannanase reduced the population of fecal coliforms and tended to reduce the NH_3_ concentration of fecal slurry after 24 h fermentation.

Generally, animals need an adequate amount of trace minerals to avoid deficiencies such as low appetite, poor growth, and diarrhea. In particular, zinc oxide becomes an innate micromineral to regulate the growth and immune function of animals (12) as it plays a vital role in growth and cell proliferation. Previously, Roselli et al. (13) reported that Zn exhibits an antibacterial agent; for example, it prevents the adhesion and internalization of enterotoxigenic *E. coli* into enterocytes. Owing to its antibacterial, disinfecting, and anti-inflammation properties, it plays an imperative role in pharmaceuticals and agriculture, also widely used as a dietary supplement (14).

**Figure 1:**
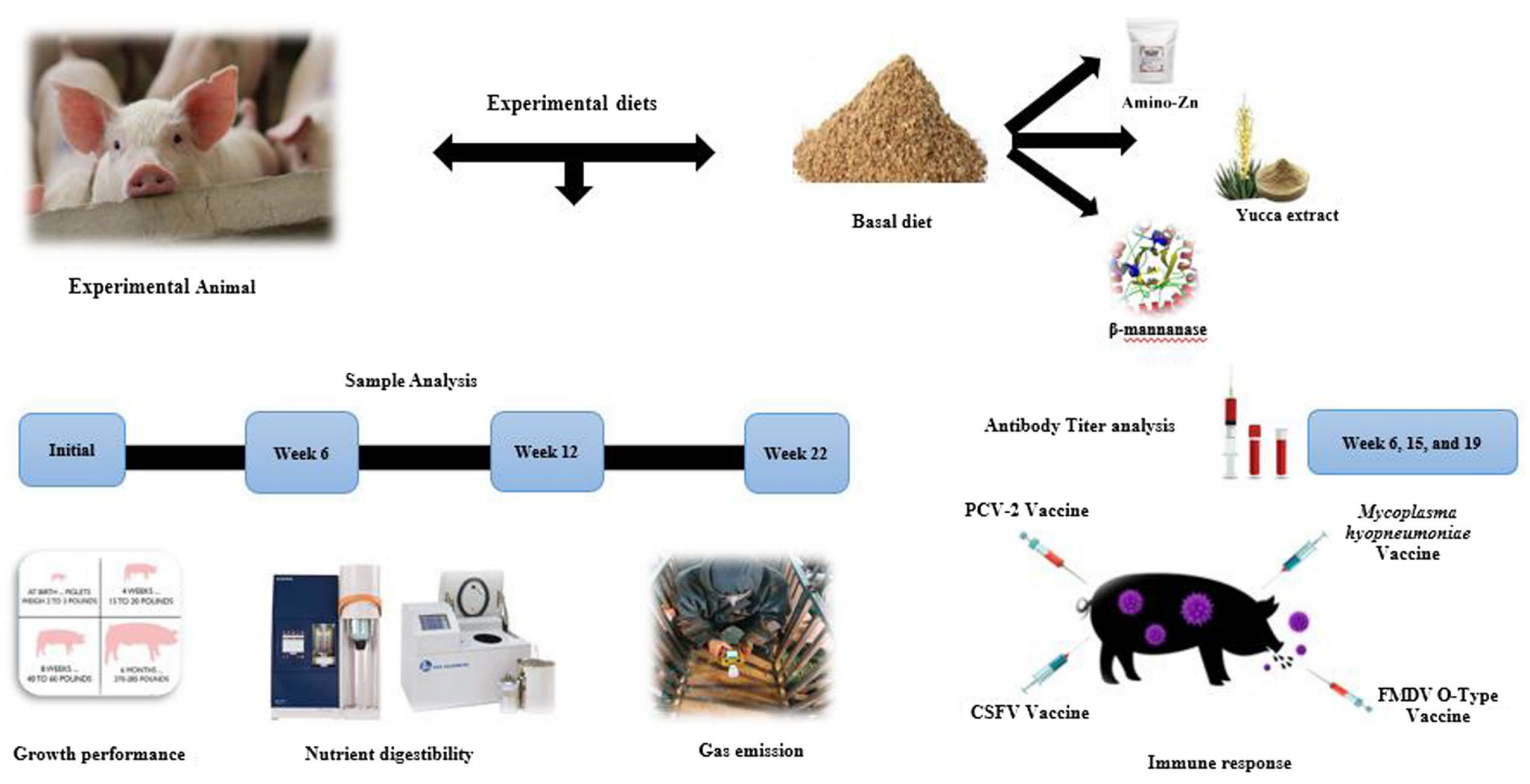
Schematic view of the experiment design.

Porcine circovirus type 2 (PVC-2) is one of the most important pathogens in the pig industry. Apart from this, foot-and-mouth disease (FMD) causes extensive financial loss and devastating effects on pig production (15). Furthermore, *Mycoplasma hyopneumoniae* (MH), a severe respiratory problem, leads to reduced weight gain and poor feed conversion in pigs (16). Previously Van Heugten et al. (17) stated that endotoxin injection helped to induce the immune response in pigs. However, Hevener et al. (18) reported that the acute effects of endotoxin treatment rarely mimic the long-term detrimental influence of infectious diseases in grow-finish pigs. To prevent severe diseases, vaccines have been applied to pigs, but those vaccines do not prevent the outbreak of diseases. Probably these vaccines have a poor cell-mediated immune (CMI) response and a short duration of immunity (19). Thus, there is a growing need to explore alternative anti-PCV, anti-FMD, and anti-*M. hyopneumoniae* agents with different mechanisms of action. In 2014, Chai et al. (20) reported that the inclusion of high levels (2,500 mg/kg) of Zn supplementation in pigs’ diets showed enhanced protection in the intestinal tract and stimulated the systemic humoral immune response against transmissible gastroenteritis viral infection. The above-mentioned literature (6, 8, 11, 20) has prompted us to hypothesize that the inclusion of AZn, YE, and β-mannanase supplements might enhance the growth performance, nutrient digestibility, and immune response and reduce fecal gas emission in pigs. To the best of our knowledge, this would be the first study to explore the efficacy of dietary AZn, YE, and β-mannanase blends on performance and antibody titers in pigs with response to vaccination (Figure 1). Thus, this study investigated the impacts of in-feed additive blends on growth performance, nutrient digestibility, fecal gas emission, and serological immune responses to swine diseases in weaning to finishing pigs.

## 2. Materials and methods

### 2.1. Ethical endorsement

Husbandry practices were strictly performed according to the guidelines of animal welfare, and the research protocol (No: DK-2-2203) was approved by the Institutional Animal Care and Use Committee (IACUC) of Dankook University (Cheonan, Republic of Korea) prior to the trial.

### 2.2. Research site and source of the main additive

This experiment was carried out at the Dankook University “Swine research unit” located in Gongju City (Republic of Korea). The main supplements AZn, *Yucca schidigera* extract, and β-mannanase enzyme (powdered form) used in this study were obtained from BTN Co. Ltd. (Asan-si, Republic of Korea).

### 2.3. Animals, trial design, and husbandry management

**Figure 2:**
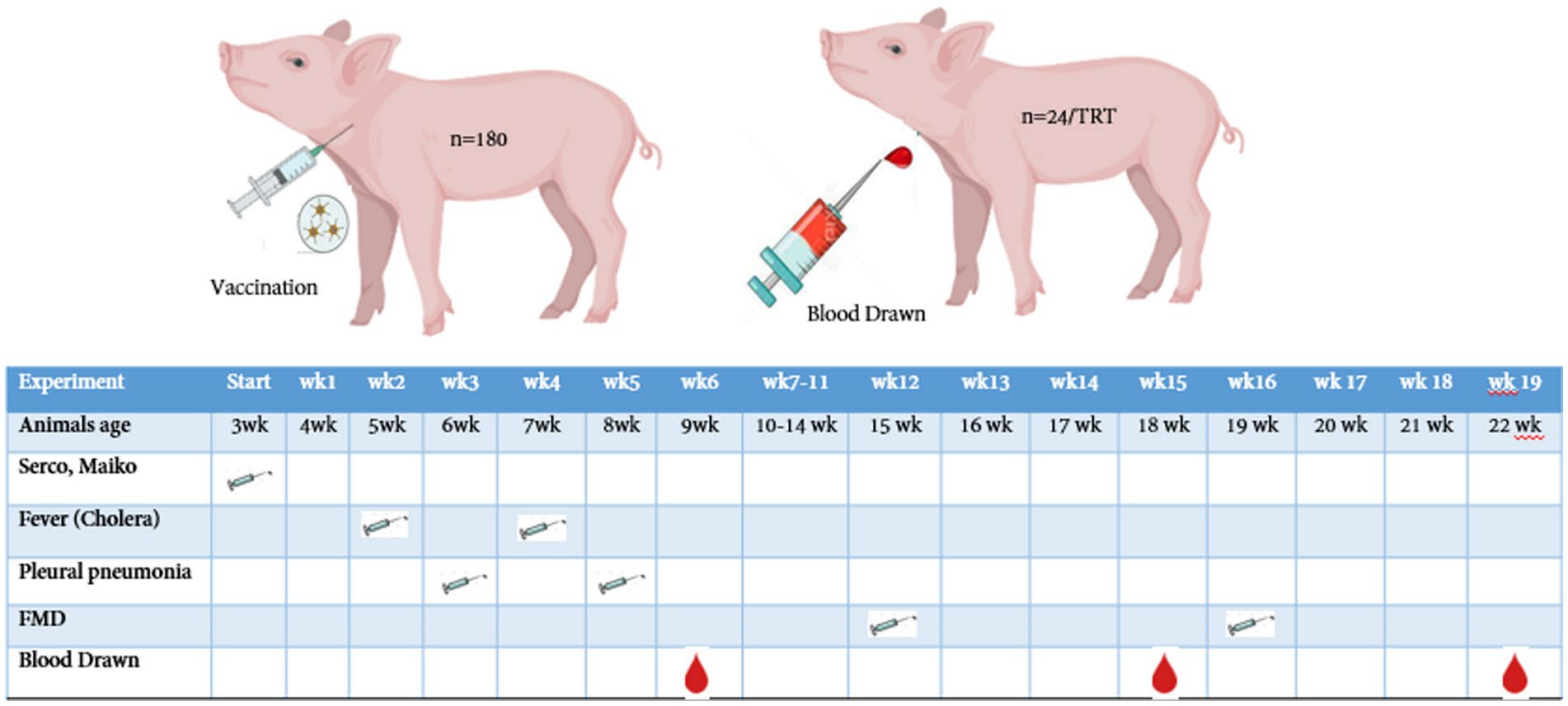
Vaccination and blood collection schedule of pigs from weaning to finishing stage. A total of 180 pigs (12 replicates × 3 treatemet, with 5 pigs/pen) were vaccinated at different periods, and the blood samples were randomly drawn from *n* = 24/treatment.

A total of 180 crossbred [(Landrace ×Yorkshire) × Duroc] pigs were used in this 22-week trial. The mean body weights of pigs for all the pens were made to be approximately 6.57 ± 1 kg. There were three treatment groups with 12 replicates with 5 pigs (2 barrows and 3 gilts) per pen. Pigs were arranged in a complete randomized block design at the weanling stage, and this arrangement was continued until the finishing stage. The weaning room temperature was set up to 33°C at first, and later it was reduced to 28°C (83°F), and 60% humidity was maintained. Meanwhile, the growing–finishing facility was equipped with natural ventilation and completely slatted concrete floors, and the room temperature was fixed at 21.5 ± 1.12°C until week 22. The bite-type nipple drinkers and commercial feeder hopper with three feeder spaces were fixed at the corner of each pen, allowing the weaning pigs to access free feed and water throughout the trial. Meanwhile, single-space feeders and water drinkers were fixed at the corner of each pen (1.8 m × 1.8 m) for the growing–finishing pigs to access *ad libitum* feed and water, respectively. The experimental animals were vaccinated with four different vaccines: porcine circovirus type 2 (PCV-2), classical swine fever virus (CSFV), *Mycoplasma hyopneumoniae* (MH), and foot-and-mouth disease-type (FMDV) at initial (3 weeks old) to 19 weeks (22 weeks old) (Figure 2). The breeding room was strictly monitored by the trainees thrice a day (9:00 AM, 2:00 PM, and 7:00 PM) to check whether the feeder had sufficient feeds, any leakage in the water trough, and occurrence of health issues.

### 2.4. Experimental diets and dietary schedule

According to initial body weight (6.57 ± 1 kg) and sex, pigs were randomly assigned into one of three dietary treatment groups, i.e., CON, TRT 1, and TRT2. The experimental diets were as follows.

1. CON-Basal diet (corn and soybean meal)
2. TRT1-CON +1,000 ppm AZn + 0.07% yucca extract (YE) + 0.05% β-mannanase
3. TRT2-CON +2,000 ppm AZn + 0.07% YE+ 0.05% β-mannanase

The basal diet (mash form) for each growth phase, i.e., weaning Phase I (weeks 0–1), Phase II (weeks 1–3), and Phase III (weeks 3–6), was formulated to meet the recommendation of NRC, 2021 (21) (Table 1), while growing (weeks 6–12) and finishing [Phase I (weeks 12–18) and Phase II (weeks 18–24)] were formulated to meet the recommendation of NRC, 1988 (22) (Table 2). The basal diet and additives were blended using a DDK-801 feed mixer (Daedong Tech, Gyeonggi-do, Republic of Korea).

**Table 1:** Ingredient composition of experimental diets on as-fed basis.

	Weaning		
Items	Phase 1	Phase 2	Phase 3
Corn	37.70	52.03	60.83
Soybean meal, 48%	18.25	16.68	19.05
Fermented SBM	5.00	4.00	3.00
Blood plasma protein	5.00	3.00	2.00
Tallow	2.90	2.69	2.25
Lactose	13.46	7.78	3.18
Sugar	3.00	3.00	3.00
Whey protein	11.00	7.00	3.00
MDCP	1.10	1.30	1.35
Limestone	0.93	0.94	1.00
Salt	0.20	0.10	0.10
Methionine (99%)	0.22	0.15	0.09
L-lysine (78%)	0.51	0.65	0.57
Vit/Min premix^a^	0.40	0.40	0.40
Choline (25%)	0.03	0.03	0.03
AZn (80%)	0.30	0.25	0.15
Total (%)	100.00	100.00	100.00
Calculated value			
CP, %	20.00	18.00	18.00
ME, kcal/kg	3,450	3,400	3,350
FAT, %	4.56	4.79	4.66
Ca, %	0.80	0.80	0.80
P, %	0.60	0.60	0.60
LYS, %	1.60	1.50	1.40
MET, %	0.48	0.40	0.35
Lactose, %	20.00	12.00	5.00

a Provided per kg of complete diet: 16,800 IU vitamin A; 2,400 IU vitamin D_3_; 108 mg vitamin E; 7.2 mg vitamin K; 18 mg Riboflavin; 80.4 mg Niacin; 2.64 mg Thiamine; 45.6 mg D-Pantothenic; 0.06 mg Cobalamine; 12 mg Cu (as CuSO4); 60 mg AZn (as amino–AZn complex); 24 mg Mn (as MnSO4); 0.6 mg I (as Ca (IO3)2); 0.36 mg Se (as Na2SeO3).

**Table 2:** Ingredient composition of experimental diets on as-fed basis.

Items	Growing	Finishing	
		Phase 1	Phase 2
Corn	37.57	37.98	36.15
Wheat	19	24	29
Rice bran	2	2	2
Wheat bran	2	-	-
Palm kernel meal	2	3	3
Soybean meal	3	3	3
Dehulled soybean meal	15.11	11.34	8.12
Rape seed meal	4	4	4
Sesame meal	2	2	2
Brown rice	5	5	5
Animal fat	3.79	3.26	2.89
Molasses	2	2	2
Limestone	1.05	1.08	1.1
MCP	0.16	0.1	0.09
Salt	0.3	0.3	0.3
Methionine 98%	0.01		0.01
Threonine 98%	0.02	0.01	0.05
Lysine 25%	0.5	0.49	0.79
Choline Chloride 50%	0.09	0.09	0.1
Mineral^a^/Vit premix^b^	0.4	0.35	0.4
Total	100.00	100.00	100.00
Calculated value			
Digestible energy (kcal/kg)	3,560	3,540	3,510
Metabolic energy (kcal/kg)	3,280	3,260	3,250
CP (%)	17.50	16.00	15.00
C. Fat (%)	6.70	5.90	5.50
C. Ash (%)	4.40	4.20	4.10
*C. fiber* (%)	3.80	3.90	3.90
Total lysine (%)	0.99	0.88	0.86
Calcium (%)	0.75	0.65	0.65
Phosphorus (%)	0.42	0.39	0.39

a Provided per kg diet: Fe, 100 mg as ferrous sulfate; Cu, 17 mg as copper sulfate; Mn, 17 mg as manganese oxide; AZn, 100 mg as zinc oxide; I, 0.5 mg as potassium iodide; and Se, 0.3 mg as sodium selenite.

b Provided per kilograms of diet: vitamin A, 10,800 IU; vitamin D3, 4,000 IU; vitamin E, 40 IU; vitamin K3, 4 mg; vitamin B1, 6 mg; vitamin B2, 12 mg; vitamin B6, 6 mg; vitamin B12, 0.05 mg; biotin, 0.2 mg; folic acid, 2 mg; niacin, 50 mg; D-calcium pantothenate, 25 mg.

### 2.5. Specimen collection and clinical analyses

#### 2.5.1. Growth performance

The growth performance variables, namely, body weight (BW), average daily gain (ADG), average daily feed intake (ADFI), and gain-to-feed ratio (G: F), were recorded at different growth stages. Individual body weight was measured using a GL-6000S machine (G-Tech Inc., LTD., Seoul, Republic of Korea) at the beginning and end of weeks 6, 12, 18, and 22 to determine their average daily gain (ADG). The feeders were filled in the morning (9:00 AM), and the remaining feeds in the feeders were collected and weighed in the evening (5:00 PM) to calculate the ADFI. The G: F was determined by dividing the ADFI and ADG.

#### 2.5.2. Nutrient digestibility

Seven days prior to the fecal collection (end of each growth stage), i.e., week 6, 12, and 22, 0.5% chromium oxide (Cr2O3) as an indigestible marker was added to the pigs’ diet in order to measure the nutrient digestibility of dry matter (DM), nitrogen (N), and gross energy (GE). After mixing the chromium oxide, the representative feed samples from each treatment group were collected and stored in sterilized plastic bags for further analysis. At the end of weeks 6, 12, and 22 (10:00 AM and 3:00 PM), approximately 200 g of fresh fecal specimens were randomly collected from two pigs per pen (one barrow and one gilt) by rectal palpation and homogenized. Within 45 min, the collected specimens were taken to the laboratory and stored at −20°C to prevent changes in nutrients. Before starting the analysis, the fecal samples were kept in the WOF-L800 hot air convection drying oven (Daehan Scientific Co. Ltd., Wonju, Republic of Korea) for 2 days at 105°C. Then, the samples were taken from the dryer and grounded well (Willey Mill, United States) to pass through a 1-mm screen sieve. Simultaneously, the feed samples were also grounded. The nutrient digestibility of DM, N, and GE was analyzed according to the AOAC guidelines (23). The chromium concentration in the sample was analyzed using UV spectrophotometry (Shimadzu, UV-1201, Kyoto, Japan), and the readings were noted. Nutrient digestibility (ND) was analyzed using the following formula: ND (%) = 100−[(NF/ND) × (CrD/CrF)] × 100]. Here NF, ND, CrD, and CrF stand for nutrient concentration in the feces sample, nutrient concentration in the diet, chromium concentration in the diet, and chromium concentration in the feces sample, respectively. The gross energy in the feed and fecal sample was measured using the Parr 6,400 (Parr instrument, Moline, IL, United States) oxygen calorimeter. The crude protein (N) levels in the feed and fecal samples were determined using the Tector Kjeltec™ 8,400 (FOSS Co. LTD., Hilleroed, Denmark) automatic analyzer.

#### 2.5.3. Fecal gas emission

At the end of weeks 6, 12, and 22, approximately 300 g of stock feces were collected (*n* = 2 pen/treatment) and placed in plastic boxes with a small hole in the middle and sealed with plaster. The samples were fermented for 1 day at room temperature (25°C), and 100 mL of the sample was taken from the headspace (approximately 2.0 cm) above the fecal sample for air circulation. Later, the box was re-sealed to measure the noxious fecal content. Before measurement, the slurry samples were manually shaken for around 30 s to measure the crust formation on the surface and homogenized. The adhesive plaster was punctured, and 100 mL of the headspace air was sampled approximately 2.0 cm above the fecal surface. NH_3_, H_2_S, methyl mercaptan, CO2, and acetic acid concentrations were measured using a Multi-RAE Lite-gas search 121 probe (model PGM-6208, RAE, United States).

#### 2.5.4. Antibody titer analyses

For the antibody titer analysis, at the end of weeks 6,15, and 19, blood samples were collected after 12 h of fasting from the jugular vein (*n* = 24 pigs/treatment) using 10 mL vacutainer glass tubes (Venoject^®^, Terumo Europe N.V., Leuven, Belgium). The collected specimens were taken to the laboratory and centrifuged at 3,000×*g* for 15 min at 4°C (centrifuge MF-550, Hanil Science Industrial Co. LTD, Incheon, Republic of Korea). Then, the plasma was carefully transferred to 1.5 mL microtubes and tested with PCV-2 (SERELISA^®^ PCV2 Ab Mono Blocking, Synbiotics Corp., Lyon, France) and CSFV (IDEXX CSFV Ag Serum Plus, IDEXX Laboratories, Inc., Maine, United States) using enzyme-linked immunosorbent assay (ELISA) kits following the manufacturer’s protocol. The MH serum samples were tested with an ELISA (DAKO *Mycoplasma hyopneumoniae* ELISA^®^; DAKO A/S, Glostrup, Denmark). The anti-FMDV serotype O antibody level of the serum sample was determined using a Prio-CHECK FMDV type O ELISA kit (Prionics, Switzerland).

#### 2.5.5. Statistical analyses

Experimental data were analyzed using the GLM procedure of SAS Inst. (Inc., Cary, NC, United States). Growth performance analysis was performed using GraphPad Prism statistical software version 5.0. Orthogonal polynomial contrasts were used to evaluate the linear and quadratic effects of dietary AZn, YE, and β-mannanase supplementation. The pen was used as the experimental unit for the analysis of growth performance, nutrient digestibility, fecal gas emission, and antibody titers. A probability value of less than 0.05 was considered significant, and less than 0.10 was considered a trend.

## 3. Results

**Figure 3:**
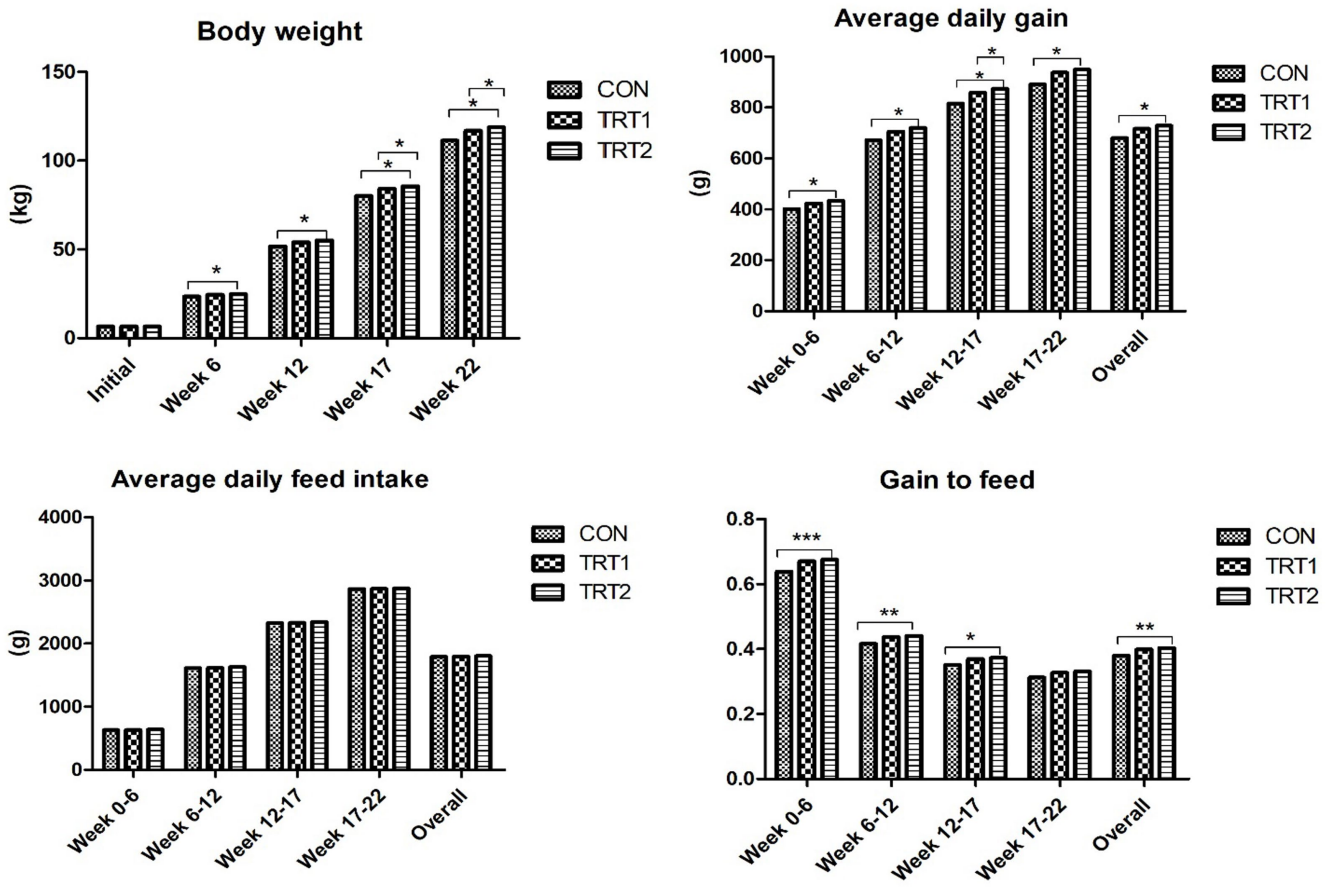
Effects of dietary AZn, *Yucca schidigera* extract, and β-mannanase on growth performance of pigs at different growth stages. Data are presented as standard means of error. *, **, and *** indicate statistically significant at *p* < 0.05, *p* < 0.001, and *p* < 0.0001, respectively.

Pigs fed diet supplements with AZn, YE, and β-mannanase linearly increased (*p* < 0.05) BW and average daily gain at weeks 6, 12, 17, and 18. Also, the gain-to-feed ratio showed a linear increase (*p* < 0.05) at the end of weeks 6, 12, 17, and the overall experimental period compared to those fed CON diet. There was no difference in feed intake (Figure 3) and nutrient digestibility of DM, N, and GE throughout the trial (Table 3). However, the inclusion of experimental diets linearly decreased (*p* < 0.05) noxious gas emissions such as NH_3_, H_2_S, acetic acid, Methyl mercaptans, and CO_2_ at the end of week 22 (Table 4). The dietary inclusion of AZn, YE, and β-mannanase significantly reduced the S/P ratio for *Mycoplasma hyopneumoniae* (MH) compared with control. However, at the end of week 6 and 19 pigs showed linearly increased (*p* < 0.05) serological immune responses for foot-and-mouth disease virus (FMDV-O type) and porcine circovirus-2 (PCV-2), respectively (Table 5).

**Table 3:** The effect of dietary AZn, *Yucca schidigera* extract, and β-mannanase supplementation on nutrient digestibility in pigs.^a^

Items, %	CON	TRT1	TRT2	SEM^b^	*p*-value	
					Linear	Quadratic
Week 6						
Dry matter	82.15	82.80	83.41	0.87	0.323	0.988
Nitrogen	80.26	80.67	81.44	0.89	0.362	0.867
Gross energy	81.2	81.75	82.54	0.92	0.323	0.917
Week 12						
Dry matter	74.07	74.61	75.17	0.98	0.443	0.994
Nitrogen	71.80	72.47	72.98	0.95	0.392	0.948
Gross energy	72.91	73.49	73.87	0.92	0.467	0.928
Week 22						
Dry matter	69.57	70.72	71.21	0.92	0.225	0.770
Nitrogen	67.66	68.89	69.58	0.91	0.155	0.810
Gross energy	68.71	69.70	70.29	0.94	0.256	0.867

a CON, Basal diet; TRT1, Basal diet +1,000 ppm AZn + 0.07% YE + 0.05% β-mannanase; TRT2, Basal diet +2,000 ppm AZn + 0.07% YE + 0.05% β-mannanase.

b Standard error of means.

**Table 4:** The effect of dietary AZn, *Yucca schidigera* extract, and β-mannanase supplementation on gas emission in pigs.^1^

Items, ppm	CON	TRT1	TRT2	SEM^2^	*p*-value^3^	
					Linear	Quadratic
Week 6						
NH_3_	5.50^a^	4.88^ab^	4.38^b^	0.22	0.013	0.821
H_2_S	5.20	4.63	4.50	0.40	0.261	0.661
Methyl mercaptans	6.13	5.25	5.50	0.76	0.582	0.567
Acetic acid	8.38	7.38	7.00	0.43	0.065	0.575
CO_2_	12,375^a^	11,050^b^	11,150^b^	115	0.001	0.002
Week 12						
NH_3_	5.25^a^	4.63^ab^	4.25^b^	0.20	0.012	0.627
H_2_S	5.03^a^	4.40^b^	4.25^b^	0.21	0.038	0.385
Methyl mercaptans	5.88	5.13	5.00	0.56	0.312	0.665
Acetic acid	7.63a	6.75^b^	6.50^b^	0.31	0.044	0.448
CO_2_	12,775^a^	10,975^b^	11,000^ab^	298	0.005	0.046
Week 22						
NH_3_	5.25^a^	4.50^b^	4.13^b^	0.19	0.005	0.453
H_2_S	5.18^a^	4.35^ab^	4.05^b^	0.31	0.041	0.511
Methyl mercaptans	6.13^a^	5.13^ab^	4.88^b^	0.34	0.042	0.407
Acetic acid	8.13^a^	6.63^b^	6.38^b^	0.40	0.021	0.248
CO_2_	12,100^a^	10,825^ab^	10,325^b^	239	0.002	0.233

1 CON, Basal diet; TRT1, Basal diet +1,000 ppm AZn + 0.07% YE + 0.05% β-mannanase; TRT2, Basal diet +2,000 ppm AZn + 0.07% YE + 0.05% β-mannanase.

2 Standard error of means.

3 Means in the same row with different superscripts differ significantly (*p* < 0.05).

**Table 5:** The effect of dietary AZn, *Yucca schidigera* extract, and β-mannanase supplementation on the immune response of pigs.^1^

Items	CON	TRT1	TRT2	SEM^2^	*p*-value^3^	
					Linear	Quadratic
**Week 6**						
**PCV-2**						
S/P ratio	1.58	1.66	1.70	0.07	0.289	0.844
Positive	100	100	100	–	–	–
**MH**						
S/P ratio	1.28^a^	0.97^b^	0.75^b^	0.15	0.048	0.817
Positive	100	100	100	–	–	–
**CSFV**						
Blocking (%)	90.29	86.77	85.59	1.48	0.065	0.543
Positive	100	100	100	–	–	–
**FMDV-O type**						
Blocking (%)	44.93^b^	69.56^a^	80.70^a^	8.33	0.022	0.533
Positive	25	75	100	–	–	–
**Week 15**						
**PCV-2**						
S/P ratio	1.38	1.52	1.54	0.09	0.267	0.632
Positive	100	100	100	–	–	–
**MH**						
S/P ratio	0.51	0.42	0.47	0.09	0.763	0.534
Positive	75	50	75	–	–	–
**CSFV**						
Blocking (%)	72.16	77.06	77.24	2.39	0.183	0.450
Positive	100	100	100	–	–	–
**FMDV-O type**						
Blocking (%)	48.39	49.08	50.11	2.40	0.630	0.955
Positive	25	50	50	–	–	–
**Week 19**						
**PCV-2**						
S/P ratio	1.48^b^	1.65^ab^	1.84^a^	0.06	0.004	0.874
Positive	100	100	100	–	–	–
**MH**						
S/P ratio	0.02	0.07	0.06	0.05	0.615	0.647
Positive	0	0	0	–	–	–
**CSFV**						
Blocking (%)	26.34	28.70	32.55	6.32	0.513	0.874
Positive	25	25	25	–	–	–
**FMDV-O type**						
Blocking (%)	12.74	19.51	22.42	4.65	0.199	0.746
Positive	0	0	0	–	–	–

1 CON, Basal diet; TRT1, Basal diet +1,000 ppm AZn + 0.07% YE + 0.05% β-mannanase; TRT2, Basal diet +2,000 ppm AZn + 0.07% YE + 0.05% β-mannanase. PCV-Porcine circovirus type 2; CSFV-Classical swine fever virus, FMDV-foot-and-mouth disease serotype O, MH-*Mycoplasma hyopneumoniae.*

2 Standard error of means.

3 Means in the same row with different superscripts differ significantly (*p* < 0.05). S/P ratio defined optical density of serum (S) /optical density of positive control (P).

## 4. Discussion

### 4.1. Growth performance

Zn at pharmacological doses (1,500–3,000 mg/kg) is widely used in the swine industry as an effective tool for managing post-weaning diarrhea (PWD), which causes significant economic losses to the production cycle of piglets (24). In addition, it exerts an optimistic effect along the total intestinal tract by targeting digestive secretions, intestinal architecture, and antioxidant systems (25). Though Zn has a moderate antibacterial effect against PWD, the environmental impact of Zn and emerging threats raise serious questions about the sustainability of its extensive utilization. Accordingly, the long-term use of Zn at the pharmaceutical level (2,500–3,000 ppm) in pigs resulted in potential toxic effects and raised several anxieties such as environment pollution, antimicrobial resistance, and modification of intestinal microbiota (26). Concerning this, the European Union (EU) has prohibited the use of Zn at the pharmaceutical level in pig production since June 2022 (24); therefore, novel strategies to manage PWD are urgently needed. Indeed, plant-derived products that contain secondary metabolites could help the animals to improve their health and production by reducing microbial resistance exhibited by pharmacological drugs (AZn) used in animal feed. Previously, Zúñiga-Serrano et al. (27) reported that secondary plant metabolites are cost-effective for improving the production efficiency of non-ruminants. Feed costs play a determining factor in profitable and sustainable animal production. In an earlier report, Kiarie et al. (28) pointed out that using exogenous enzymes (β-mannanase) has gained wide acceptance in feed cost management through the application of resourceful feed ingredients and the reduced environmental impact in animal production. Taking all the aspects into consideration, we have initiated this research. As anticipated, the current research reveals linearly increased (*p* < 0.05) BW, ADG, and gain-to-feed ratio in pigs fed a diet supplemented with AZn, YE, and β-mannanase blends. The present finding is in partial agreement with Case and Carlson (29), who noted greater ADG in weaning pigs fed a diet supplemented with 2,000–3,000 ppm of Zn. Moreover, Hong et al. (6) reported that increased (120 ppm) YE concentration in pigs’ diets improved daily feed intake and gain-to-feed ratio.

Similarly, Bae et al. (30) stated that pigs fed the yucca extract (125 ppm) diet significantly improved average daily gain. Moreover, Lv et al. (31) pointed out that adding 600 U/kg β-mannanase to the diet of growing pigs increased daily weight gain (quadratic effect; *p* < 0.01) and feed efficiency (*p* < 0.01) during the second phase and the overall experimental period. In addition, Pettey et al. (9) noted a 3.4% increased weight gain and 3.9% feed efficiency in growing–finishing pigs fed diets supplemented with β-mannanase. On the contrary, Holen et al. (32) reported that growing–finishing pigs receiving 60 to 140 mg/kg of organic and inorganic AZn sources had no impact on growth performance. Furthermore, Patience et al. (33) found no differences in growing–finishing pigs’ growth parameters with the inclusion of 50 mg/kg of AZn to lysine to calorie ratios. Yen and Pond (34) and Gipp et al. (35) also stated that yucca extract additions had no effects on average daily gain, average daily feed intake, and gain/feed in pigs, and this finding partially agreed with the present results in which pigs fed dietary supplement with AZn, YE, and β-mannanase showed no effect on their daily intake. Overall, there is little evidence in the literature to support differences in growth performance using these three additive blends in the pig’s diet; thus, we made the majority of comparisons with AZn, YE, and β-mannanase individually. Together, we speculated that the inclusion of experimental diets might satisfy the nutritional requirements of pigs to reveal better growth performance, i.e., increased body weight, daily gain, and gain-to-feed ratio. As a natural feed additive, *Yucca schidigera* (YS) has been shown to have a positive effect on promoting animal growth and improving feed utilization in livestock (36). In the current study, pigs fed YE failed to enhance the feed intake, yet this null effect is consistent with Gurbuz et al. (37) who also observed this in laying hens fed *Yucca schidigera* extract (YSE). However, the additive blends increased the daily gain and feed conversion efficiency, and this is consistent with the studies on the effects of YSE on broiler chickens, which showed that extracts of YS plants increased the daily gain and feed conversion efficiency (38). It is not known which phytochemicals of YS are responsible for the decreased nutrient utilization, but we supposed that active groups of phytochemicals contained in YS might be one of the reasons for the reduction of nutrient absorption in the digestive tract that resulted in no improvement in feed intake; thus, further study is needed to understand the exact cause for the lack of this result.

### 4.2. Nutrient digestibility

A great deal of research on pig nutrition has focused on the utilization of different in-feed additives. Still, no work has been carried out on pigs using a combination of AZn, YE, and β-mannanase supplements. Enzyme supplementation with β-mannanase to degrade β-mannan fibers in the diet has been shown to improve swine performance (39) and upregulate the metabolic functions related to digestion, metabolism, and immunity in broilers (40). In an earlier study, Qian et al. (41) reported that pigs fed a diet supplement with chitosan–Zn chelates had higher DM. However, Cho et al. (42) reported that the apparent total tract digestibility of DM, N, and GE was unaffected by dietary Zn, which was consistent with the present findings. Furthermore, Hong (6) reported that the digestibility of DM and N was not affected by the YE supplementation. In contrast, Jo et al. (10) and Kim et al. (43) reported that dietary supplements with β-mannanase or enzyme complex with β-mannanase improved the nutrient digestibility of growing pigs. The probable differences in the magnitude of the response to enzyme supplementation in the current experiment and previous results could be due to the alterations in enzyme purity, level of supplementation, thermostability, and the pH optimum of the various products used. Previously, the study of Hedemann et al. (44) demonstrated that the inclusion of 2,500 ppm Zn in weaned pigs’ diet increased the activity of several pancreatic digestive enzymes such as carboxypeptidases, trypsin, and chymotrypsin, guaranteeing a higher nutrient digestibility; in this sense, we supposed that the inclusion of AZn might not be sufficient to increase the activity of pancreatic digestive enzymes to enhance nutrient absorption. Moreover, we speculated that the direct action of this combined supplementation on digestive physiology, regulation of the intestinal development, and remodeling of the gut microbiota might not be closely associated to improved digestibility. The non-starch polysaccharides (NSP, β-mannan) content in the swine diet could depress lipid metabolism by the inhibition of lipolysis and nutrient absorption (45). From this, we speculated that NSP could impair the diffusion and connective transport of lipase, oil, and bile salt micelles within the gastrointestinal contents, leading to a change in the viscosity of the digesta and thus resulting in decreased digestibility.

### 4.3. Fecal gas emission

Ammonia emissions from animal facilities are frequently subjected to odor complaints and public anxiety due to the possibility of their adverse health effects on both humans and animals. Prolonged exposure to high ammonia levels may also lead to various disorders (46). Therefore, it is imperative to develop efficient strategies to reduce noxious emissions from livestock manure. Zn is related to considerable risks to the environment, deriving from the application of Zn-rich manure to land: manure comes from facilities employing pharmacological doses of Zn (47). Previously, Zhang et al. (48) reported that dietary supplements with 0.1% chelate copper and Zn showed no difference in fecal noxious gas production in weaning pigs. However, in this study, 0.2% of Zn combined with YE and β-mannanase highly suppressed the odor emission. The rationale behind the decreased gas emission in pigs is attributed to the inclusion of Zn, which enhances the intestinal antioxidant capacity and reduces the intestinal cell apoptotic index, thus resulting in decreased noxious gas production. In an earlier study, Cha et al. (49) reported that ammonia concentration in feces was significantly reduced by YE (0.02%) addition. Similarly, Colina et al. (50) noted that pigs fed 125 ppm of YE reduced aerial ammonia concentration by 22%. Moreover, Sutton et al. (51) pronounced that ammonia emission was significantly suppressed by 55.5% in manure by the inclusion of saponin extract. However, Upadhaya et al. (11) reported that β-mannanase supplementation to corn–SBM diets tended to reduce the NH_3_ concentration of fecal slurry after 24 h fermentation. On the other hand, Yeo and Kim (52) noted a tendency to decrease ammonia emission in broilers, while Rowland et al. (53) pointed out that yucca extract significantly reduced ammonia release in pig feces. YE has been shown to improve livestock and poultry performance, thereby increasing their daily weight gain and feed efficiency (5, 54), reducing ammonia emissions from animal manure, and consequently controlling the odor emitted from animal facilities (55). The possible reason behind the reduction of harmful gas emissions in this study could be due to the beneficial effect of the YE supplement, whose saponin fractions inhibit the activity of urease in the decomposition of urea nitrogen into ammonia, and/or due to the presence of a glycoprotein and the ammonia-binding properties of YE, which play an important role in reducing ammonia release from animal excretions, helping animals to improve their performance.

### 4.4. Vaccine-induced antibody titer

Antibodies of maternal origin are normally present in all piglets due to the adequate intake of colostrum (56), but these antibodies in serum gradually decrease during the lactation and rearing stages (57). Porcine circovirus-2 (PCV-2) usually appears between the final phase of the nursery and the start of the fattening phase (58). Furthermore, foot-and-mouth disease (FMD), an acute infectious disease caused by an RNA virus belonging to *Picornaviridae* family, which affects 77% of the global livestock population (59), and classical swine fever (CSF), a highly contagious disease caused by the classical swine fever virus (CSFV), bring huge economic loss worldwide (60). The immunologic mechanisms whereby zinc modulates increased susceptibility to infection have been studied for several decades. For instance, Mishra et al. (14) stated that zinc plays an important role in controlling the immune system, particularly in zinc-deficient animals who experience increased susceptibility to various pathogens. In addition, Saura and Crowe (61) reported that zinc has broad-spectrum anti-viral activity against a variety of viruses, such as transmissible gastroenteritis virus (TGEV), equine arteritis virus, and severe acute respiratory syndrome coronavirus. Many potential mechanisms have been suggested to explain the beneficial effect of Zn against virus infections. Overbeck et al. (62) demonstrated that Zn contributes to initiating and maintaining robust immune responses, thereby producing cytokine and modulating the activity of immune cells. Correspondingly, Martin et al. (63) demonstrated that high levels of dietary Zn provided as Zn outbalanced Zn homeostasis with increased accumulation of Zn in various organs, including the small intestine of piglets. In recent years, herbal immune modulators have been considered safe alternatives, and they are totally free from animal or human pathogens and have been reported to show some beneficial effects in terms of cost, distribution, and production (64). Moreover, exogenous β-mannanase enzymes have been developed to inhibit innate immune stimulators in pigs (65). Here, the effect of AZn, YE, and β-mannanase supplement on the serological immune response of PCV-2, CSFV, MH, and FMDV stereotype O vaccination was studied in pigs of different ages; as a result, a significant increase of the antibody level specific to FMD O type and a tendency of increase of the antibody level specific to CSFV were observed in their serum samples at week 6. This result was constant with that of Kar et al. (66) who observed similar effects in Tamworth and Desi crossbred piglets after they were fed a diet containing 150 ppm of Zn sulfate for 60 days from 1 month of age to vaccination at 56 days of age with a cell culture-adapted lapinized CSFV strain. A study by Fu et al. (67) demonstrated the effectiveness of a Chinese herbal extract made from licorice, luhanguo, chrysanthemum, and Chinese tea in inhibiting the FMD virus in suckling mice. Furthermore, experimental diets significantly increased the antibody level in pigs at the end of week 19 and suppressed the shedding of PCV-2. Previously, Zhao et al. (68) reported on zinc-deficient states, a reduced immune response to hepatitis B vaccination in offspring mice, suggesting a possible enhancement of antibody formation by zinc supplementation (69). Though Zn, YE, and β-mannanase additives showed certain benefits for pig health and performance in earlier studies, to date their direct impact on antibody production against various virus is not well elucidated. Herein, we speculated that timely vaccination with appropriate vaccines targeting these specific viruses along with experimental diets might prevent them from shedding FMDV type O and PCV-2 viruses. Therefore, the influence of these stimulants needs further trials to discern the pertaining mechanism of action on these supplement blends.

## 5. Conclusion

Our study demonstrates that the inclusion of 2,000 ppm of AZn, 0.07% of yucca extract, and 0.05% of β-mannanase enzyme supplement blends could be beneficial in decreasing noxious gas production and increasing the growth performance (BW, ADG, G: F) of pigs without adverse effects on nutrient digestibility. Moreover, adding these three additive blends to pigs’ diets along with timely vaccination could significantly reduce the S/P ratio for *Mycoplasma hyopneumoniae* and increase the serological immune responses to foot-and-mouth disease virus and porcine circovirus-2. Based on this result, we infer that a blend of AZn, YE, and β-mannanase would serve as a novel in-feed additive to enhance the performance of pigs. It also acts as a boosting agent and immune stimulatory to increase the efficacy of swine vaccination.

## Data availability statement

The original contributions presented in the study are included in the article/supplementary materials, further inquiries can be directed to the corresponding author.

## Ethics statement

The animal study was reviewed and approved by the Experimental was conducted with strict guidelines of the Institutional Animal care and use Committee (IACUC), and the research protocol (DK-2-2203) was approved by Dankook University (Cheonan, Republic of Korea), prior to the trial.

## Author contributions

BRL and IK: conceptualization. VS, NHK, and SC: methodology. VS, BRL, and NHK: formal analysis. VS and SC: writing-original draft preparation. IK: investigation, reviewing, and editing. All authors contributed to the article and approved the submitted version.

## Funding

This work was supported by the International Science & Business Belt support program, through the Korea Innovation Foundation funded by the Ministry of Science and ICT(1711150853).

## Conflict of interest

Authors BRL and NHK were employed by ZinexBio Corporation.

The remaining authors declare that the research was conducted in the absence of any commercial or financial relationships that could be construed as a potential conflict of interest.

## Publisher’s note

All claims expressed in this article are solely those of the authors and do not necessarily represent those of their affiliated organizations, or those of the publisher, the editors and the reviewers. Any product that may be evaluated in this article, or claim that may be made by its manufacturer, is not guaranteed or endorsed by the publisher.
